# Cogent integration of inflammatory biomarkers and perioperative complications of thyroid surgery in thyroidology

**DOI:** 10.1590/1806-9282.20240378

**Published:** 2024-08-16

**Authors:** Azime Bulut, Ilker Sengul, Demet Sengul, Fatma Alkan Bayburt, Esma Cinar

**Affiliations:** 1 Giresun University, Faculty of Medicine, Department of Anesthesiology and Reanimation – Giresun, Turkey.; 2 Giresun University, Faculty of Medicine, Division of Endocrine Surgery – Giresun, Turkey.; 3 Giresun University, Faculty of Medicine, Department of General Surgery – Giresun, Turkey.; 4 Giresun University, Faculty of Medicine, Department of Pathology – Giresun, Turkey.; 5 Giresun Education and Research Hospital, Department of Anesthesiology and Reanimation – Giresun, Turkey.

**Keywords:** Inflammation, Biomarker, Pathology, Thyroid gland, Thyroidology, Thyroidologists

## Abstract

**OBJECTIVE:**

Back to the sources, postoperative nausea and vomiting, hypo- and hypertension, heart rate alterations, and hypoxemia due to laryngospasm might be considered perioperative complications.

**METHODS:**

This cross-sectional study was conducted at an Education and Research Hospital between January 2018 and June 2023. The study included a total of 437 cases of thyroid surgery. The demographic data such as age, sex, co-morbidities of the instances, hypotension, hypertension, bradycardia, hypoxemia, and postoperative nausea and vomiting, as well as laboratory data were obtained and analyzed.

**RESULTS:**

Of 437 cases, 334 (76%) were females and 103 (24%) were males, with a mean age of 51.83±11.91 years and 55.32±11.87 years, respectively. No statistical significance was realized between the complications, co-morbid diseases, and age. Notably, no liaison between the complications after awakening from the anesthesia and preoperative laboratory parameters was discerned. However, a high but no significant relationship was revealed between the platelet-to-lymphocyte ratio (P/L) in cases with hypoxemia and hypotension. Finally, no significance between laboratory values, bradycardia, hypertension, and postoperative nausea and vomiting was distinguished.

**CONCLUSION:**

We postulate that the so-called inflammatory biomarkers measured at the time of preoperative examination in the blood count concept selectively do not enrich for anticipating complications that arise in the perioperative echelon.

## INTRODUCTION

Thyroidectomy is the most common endocrine surgery that is carried out globally. Though thyroid surgery is considered to have low morbidity and mortality, it is still essential to predict and attenuate relevant perioperative complications. Postoperative nausea and vomiting (poNV), hypotension and hypertension, heart rate alterations, and hypoxemia due to laryngospasm might be considered perioperative complications. Moreover, increased mean platelet volume (MPV) value has been enunciated to be associated with situations such as atherosclerosis, cardiac diseases, and obstructive sleep apnea disorders^1^. Some authors propounded a liaison between MPV values and hypertension as well as the development of preeclampsia during pregnancy^2,3^. The undulations in blood pressure and heart rate during the perioperative period more frequently emerge in cases with cardiac comorbidities. However, augmented MPV was raised in cardiovascular disorders, cerebral stroke, respiratory diseases, chronic renal failure, diabetes, and various carcinomas^4^.

Inhaled anesthetics, nitrous oxide, and opioids can be counted as risk factors for poNV, which is a frequent complication of general anesthesia that leads to discomfort for the patient. poNV remains a common issue for many cases, particularly those regarded as high risks, such as women, non-smokers, and individuals with a history of motion sickness, despite the numerous recommendations and strategies aimed at reducing it^5^. Studies suggesting that platelet-to-lymphocyte ratio (P/L) and neutrophil-to-lymphocyte ratio (N/L) could serve as indicators for poNV risk often draw on the idea that inflammation^6–9^ may play a role in its development. Inflammation can influence various physiological processes, including those related to the gastrointestinal system and vomiting behavior^6^. Many studies revealed that P/L and N/L are essential indicators of systemic inflammation, and their values with mortality, morbidity, prognosis, and surgical complications have been enunciated^6–11^. This study aimed to investigate whether preoperative MPV, P/L, or N/L was an indicator of complications and identify its relationship with the need for treatment.

## METHODS

### Study design

This study was conducted according to the declaration of Helsinki. This cross-sectional study was conducted at the Giresun Education and Research Hospital, Giresun, Turkey, between January 2018 and June 2023. The study incorporated a total of 437 cases who had undergone thyroid surgery. The inclusion criteria were (i) being over 18 years old, (ii) possessing an American Society of Anesthesiologists Physical Status I–III, and (iii) undergoing thyroid surgery. The demographic data such as age, sex, and co-morbidities of the cases were obtained. We examined anesthesia follow-up papers and recovery room records for hypotension, hypertension, bradycardia, hypoxemia, and poNV.

### Laboratory parameters

All the cases were examined preoperatively with blood count samples. The relevant white blood cells, neutrophils, lymphocytes, platelets, hemoglobin, MPV, N/L, and P/L were recorded meticulously.

### Statistical analysis

The sample size of our study was calculated using a one-way analysis of variance (ANOVA) experimental design, taking power (power of the test) 0.80, effect size 0.35, and type-1 error 0.05, and it was determined as "a total of 96 samples/patients with a minimum of 24 samples in each group." The study used Kolmogorov-Smirnov (n>50) and skewness-kurtosis tests to check whether the measurements were continuous. The descriptive statistics of this study were expressed as mean, standard deviation, minimum, maximum, number (n), and percent (%). Independent t-test and one-way ANOVA were used to compare the measurements according to the categorical groups. Kruskal-Wallis and Mann-Whitney U tests compared the non-normally distributed data. Furthermore, we used the Duncan test to identify different groups following variance analysis. We calculated Pearson correlation coefficients to determine the relationship between measurements. Moreover, we used the chi-square (Fisher's exact) test to determine the relationships between categorical variables. Statistical significance level (a) was taken as 5% (95% confidence interval) in the calculations, and the SPSS (IBM for Windows, v.26) statistical package was used for analysis. Binary logistic regression analysis was applied to describe the effect of "MPV, P/L, and N/L" on "the presence of hypoxia."

## RESULTS

In all, 437 patients undergoing thyroid surgery were included in the analysis. The patients’ characteristics and postoperative complications are presented in Table 1. As such, 334 (76%) were females and 103 (24%) were males. The mean age was 51.83±11.91 years in females and 55.32±11.87 years in males. There was no significant relationship between the age and gender of the patients included in the study (p>0.001). The mean ages of the female and male groups were similar. The demographics and clinical characteristics of the matched patient pairs are summarized in Table 1.

**Table 1:** The demographic and categorical characteristics.

		n	%
Sex	Female	334	76
	Male	103	24
Diabetes mellitus		33	7.6
Hypertension		135	30.9
Cardiac diseases		7	1.6
COPD		8	1.8
Hypothyroidism		264	60.4
Complications	Desaturation	151	34.7
	Bradycardia	53	12.2
	Hypotension	35	8
	Hypertension	3	0.7
	PONV	188	43.2

poNV: postoperative nausea and vomiting.

When the relationship between systemic diseases and patient characteristics was examined, a significant association was found between hypertension and age (p<0.001). The cases with hypertension had a noticeably higher age. No statistically significant difference between sex and systemic diseases was recognized, whereas the frequency of COPD was higher in males. In addition, no considerable difference (p>0.05) in the comparison of other systemic diseases and laboratory values was raised (Table 2). A weak significance was revealed between age and platelets (PLT), hemoglobin (Hgb), and MPV. There was no significant difference between sex and laboratory parameters, but WBC was significantly higher in males only. The overall incidence of the complications was 69%. Patients who developed hypotension had a significantly higher average age. However, no significant relationship was recognized between complications and other systemic diseases and age.

**Table 2:** The association between comorbidities and adverse events.

		Desaturation	Bradycardia	Hypotension	Hypertension	pONV
Diabetes mellitus (n=33)	Yes (n)	10	3	5	0	13
	p-values	0.716^a^	1.000^a^	0.170^a^	1.000^a^	0.781^a^
Hypertension (n=135)	Yes (n)	43	16	7	1	61
	p-values	0.400^a^	1.000^a^	0.200^a^	1.000^a^	0.579^a^
Cardiac diseases (n=7)	Yes (n)	1	1	0	2	0
	p-values	0.689^a^	0.205^a^	1.000^a^	1.000^a^	1.000^a^
COPD (n=8)	Yes (n)	3	3	2	0	3
	p-values	1.000^a^	1.000^a^	0.130^a^	1.000^a^	1.000^a^
Hypothyroidism (n=264)	Yes (n)	94	107	19	39	2
	p-values	0.577^a^	0.053^a^	0.549^a^	1.000^a^	0.187^a^

a Chi-square independence tests. COPD: chronic obstructive pulmonary disease. poNV: postoperative nausea and vomiting.

The association between complications occurring after awakening from anesthesia and preoperative laboratory parameters, a high P/L in cases with hypoxemia and hypotension, emerged without any significance (Table 3). Finally, there is no statistical significance in laboratory values for bradycardia, hypertension, and poNV.

**Table 3:** The association between laboratory parameters and anesthetic complications.

		Desaturation	Bradycardia	Hypotension	Hypertension	pONV
Leukocyte	Mean±SD	7529 (2.30)	7.133 (1.88)	7.590 (1.96)	9.576 (2.95)	7.379 (2.1)
	p-value	0.992^b^	0.235^b^	0.671^b^	0.102^b^	0.292^b^
Neutrophil	Mean±SD	4480 (1.85)	4135 (1.55)	4.329 (1.57)	6003 (3.63)	4391 (1.7)
	p-value	0.970^b^	0.075^b^	0.860^b^	0.484^b^	0.154^b^
Platelets	Mean±SD	257.33 (66.7)	246.37 (56.7)	265.28 (70.5)	352.3 (10.4)	264.27 (60)
	p-value	0.103^b^	0.038* b	0.969^b^	0.015* b	0.356^b^
Lymphocyte	Mean±SD	2328 (0.71)	2220 (0.61)	2599 (0.73)	2803 (0.5)	2282 (0.6)
	p-value	0.485^b^	0.412^b^	0.006** b	0.129^b^	0.412^b^
Hemoglobin	Mean±SD	13.51 (1.5)	13.71 (1.3)	13.91 (1.2)	12.80 (0.45)	13.50 (1.52)
	p-value	0.784^b^	0.149^b^	0.062^b^	0.262^b^	0.881^b^
MPV	Mean±SD	9.75 (1.04)	9.51 (1.10)	9.50 (1.04)	10.03 (0.1)	9.54 (1.1)
	p-value	0.028* b	0.414^b^	0.825^b^	0.312^b^	0.196^b^
N/L	Mean±SD	2.07 (1.01)	1.99 (0.94)	1.79 (0.74)	2.33 (1.82)	2.07 (1.0)
	p-value	0.838^b^	0.460^b^	0.115^b^	0.704^b^	0.413^b^
P/L	Mean±SD	118.57 (45.9)	117.83 (38.3)	107.30 (33.9)	128.55 (23.9)	124.15 (42)
	p-value	0.008** b	0.418^b^	0.006** b	0.532^b^	0.514^b^

b Mann-Whitney U test

* (p<0.05;

** p<0.01).

poNV: postoperative nausea and vomiting.

## DISCUSSION

About 310 million patients undergo surgery worldwide each year^11^. Postoperative complications, such as infectious and cardiopulmonary, occur in up to 20% of patients^12^. They increase treatment costs and cause a decrease in life expectancy and quality of life. Bleeding, laryngeal nerve injury, laryngomalacia, and sore throat are complications expected after thyroid surgery. Hence, the patient should be extubated carefully due to the possibility of bleeding. This study investigated the relationship between preoperative blood count parameters and perioperative complications, and statistically significant findings were obtained. It was found that chronic obstructive pulmonary disease (COPD) is more prevalent in male patients, and those who develop hypotension tend to be older. A higher P/L was recognized in cases experiencing hypoxemia and hypotension without significance. This study evaluated the role of N/L, P/L, and MPV as predictive tools for early complications during awakening from anesthesia in thyroidectomy. Laryngospasm, hypertension, hypotension, bradycardia, and poNV are essential complications that may occur during the postoperative period. However, early detection, rapid intervention, and standardized care are crucial for successful management. Laryngospasm can become an anesthetic emergency that happens during the induction, maintenance, and emergence phases of general anesthesia. It usually manifests with the sign of inspiratory stridor, which may progress to complete obstruction, increased respiratory effort, and oxygen desaturation with or without bradycardia. This condition more frequently happens in pediatric cases, whereas its incidence was reported to be 0.78–0.94% in adults^13^. Triggering factors for that phenomenon are the inappropriate depth of anesthesia, frequent suction catheter, inhalational-induced irritation, secretion, airway stimulation, endotracheal intubation, and upper respiratory tract infection. Furthermore, tonsillectomy, adenoidectomy, appendectomy, and thyroidectomy can be considered surgical factors for laryngospasm. It can also cause negative pulmonary pressure edema (NPPE) with a significant negative intrathoracic pressure generated by forced inspiration against an obstructed airway. NPPE is a rare but life-threatening complication of general anesthesia^14^. This study revealed that P/L values were significantly high in cases with hypoxia. Nevertheless, this has led us to think the inflammation process may contribute to early postoperative complications. As such, we postulate that the so-called P/R value might guide estimating postoperative laryngospasm. Therefore, evaluating the association between inflammatory biomarkers and early postoperative anesthesia complications in a prospective-randomized trial would be appropriate. Herein, poNV is a frequent complication of general anesthesia, with a frequency of approximately 30% in the general population, and causes discomfort to the patient, which may be an obstacle to early recovery^15^. Therefore, numerous studies have focused on risk factors such as inhaled anesthetics, nitrous oxide, and opioids to be able to attenuate poNV. It might be more stressful than postoperative pain for the patients, which makes it crucial to predict and manage poNV for the comfort of the patients. In a study, it was stated that there is a relationship between the P/L and hyperemesis gravidarum^16^. A Turkish study investigated the liaison between N/L and poNV in cases who underwent maxillofacial surgery and finally reported that poNV risk augmented significantly in patients with a higher N/L. The authors claimed that antiemetic prophylaxis could be given according to the N/L value by stating that it might indicate poNV^17^. Nevertheless, this study indicates no significant association between poNV ratios and N/L values.

Cardiovascular complications, particularly hypertension, are a dime in a dozen during tracheal extubation. Patients with cardiac co-morbidities are vulnerable to hyper- or hypotension and heart rate abnormalities. Preoperative identification of high-risk individuals and appropriate perioperative management can attenuate cardiovascular risk. Furthermore, the N/L and P/L were also found to be an indicator of the prognosis in cardiovascular diseases, malignancies, and chronic inflammatory diseases^18^. Elevated MPV is associated with an increased incidence of hypertension independent of other risk factors, which suggests that platelet activity may play a role in hypertension incidence. Moreover, the P/L values were high in the patients with hypotension without a significant difference between P/L and hypotension.

We postulate that the so-called inflammatory biomarkers might not be practical for use in forecasting cardiac and respiratory complications and poNV in the perioperative period. Although many studies have shown the interrelation between these complications and biomarkers, the discrepant cutoff values in each clinical situation make these biomarkers impractical to apply in the clinical practice of the providers, such as in thyroidology, which is a crucial and pivotal field, interconnecting many organ systems of human being^18–20^. Actually, thyroidologists are defined as the "first string" players in awareness efforts globally by the 2023 American Thyroid Association^21–23^.

### Limitations

The retrospective design of this study imposes limitations on our ability to analyze the extent of saturation drops following laryngospasm and bronchospasm, whether hypertensive patients had regular blood pressure before, and all other parameters influencing these relevant complications.

## CONCLUSION

NLR, P/L, and MPV measured during preoperative examination are insufficient for predicting complications occurring in the perioperative period. To universally accept these biomarkers as predictors of risk factors, prospective studies with larger sample sizes are required, and efforts should be made to minimize conditions by thyroid providers, in particular, that can affect blood cell counts. This issue merits further investigation. Thyroidologists are defined as the "first string" players in awareness efforts globally by the American Thyroid Association in 2023. Herewith, without which, not, no overlook thyroid diseases to opt for "thyroid health" purposes.
